# Association of cardiovascular metabolic risk factor measurements with psychiatric readmission among in-hospital patients with severe mental illness: a retrospective study

**DOI:** 10.1186/s12888-022-03704-w

**Published:** 2022-01-18

**Authors:** Xiao Wei Tan, Christopher Yi Wen Chan, Alvin Wai Mum Lum, Eng Sing Lee, Yee Ming Mok, Daniel Shuen Sheng Fung, Phern Chern Tor

**Affiliations:** 1 Department of Mood and Anxiety, Institute of Mental Health, Buangkok Green Medical Park, 10 Buangkok View, Singapore, 539747 Singapore; 2 Medical Care Service, Institute of Mental Health, Singapore, 539747 Singapore; 3 Clinical Research Unit, National Healthcare Group Polyclinics, Singapore, 138543 Singapore; 4 Lee Kong Chian School of Medicine, Nanyang Technology University of Singapore, Singapore, 308232 Singapore; 5 Duke-NUS Graduate Medical School, Singapore, 169857 Singapore

**Keywords:** Severe mental illness, Cardiovascular disease, Mental-physical comorbidity, Psychiatric readmission, Metabolic risk factor

## Abstract

**Background:**

Patients with severe mental illness (SMI) and comorbid physical conditions were often associated with higher risks of mortality and hospital readmission. In this study, we aim to examine the association of cardiovascular metabolic risk factor measurements with risks of psychiatric readmissions among in-hospital patients with severe mental illness (SMI).

**Methods:**

We collected the longitudinal information of laboratory investigations, blood pressure and body mass index (BMI) among in-hospital patients who had been diagnosed with schizophrenia, major depression disorder or bipolar disorder and with comorbid diagnosis of hypertension, hyperlipidemia or diabetes from Jan 2014 to Jan 2019. The primary outcome was time to first psychiatric readmission. Cox proportional hazard model was utilized to calculate the hazard risks (HR) of cardiovascular metabolic risk factors with psychiatric readmission.

**Results:**

A total of 5,256 patients were included in the analysis. Compared to patients with normal blood parameters, patients with aberrant tests of high-density dyslipidemia (HDL) and diastolic blood pressure (DBP) during in-hospitalization period were associated with higher risks to first psychiatric readmission [ HR (Hazard Ratio), 1.37 95% Confidence interval (CI), 1.03–1.83 for HDL and HR, 1.32 (95% CI, 1.04–1.67])for DBP]. Compared to patients with optimal monitoring, patients with suboptimal monitoring of blood lipids and blood pressure during in-hospitalization period or recommended window period of cardiovascular disease (CVD) risk management were associated with higher risks to first psychiatric readmission.

**Conclusions:**

Aberrant cardiovascular metabolic blood test and blood pressure and missing measurements among in-hospital patients with SMI were associated with increased risks of psychiatric readmissions. This calls for more active screening and monitoring of CVD risk factors for those in-hospital patients in need.

**Supplementary Information:**

The online version contains supplementary material available at 10.1186/s12888-022-03704-w.

## Introduction

Severe mental illness (SMI) including schizophrenia, major depression disorder (MDD) and bipolar disorder have heavy global burdens of illness due to poor prognosis, [1–3] long-term disability, [4] increased sickness absence [4] and repeated hospitalization [5]. Patients with SMI were often associated with high risks of comorbid physical illness including cardiovascular disease (CVD) or diabetes mellitus (DM), which resulted in a significant higher incidence of adverse cardiovascular events and a shortened life span of 10 to 20 years compared with the general population [6–9]. This mortality gap has widened in recent years, even in countries where the quality of the health care system is generally acknowledged to be good [10–13]. The modifiable risk factors for comorbid CVDs include smoking, diet, exercise, obesity, hypertension, elevated blood glucose and dyslipidemia [14]. Concurrent elevation of these risk factors is more common among patients with SMI compared with the general population [15]. Developing metabolic syndrome is also a common side effect due to either typical or atypical usage of antipsychotic medications [16–18].

Various educational modules, monitoring and treatment guidelines as well as recommendations at the health care institutions and individual level have been discussed to guide the management of cardiovascular metabolic risks among patients with SMI [19–22]. Patients need be assessed at regular intervals for personal and family history of DM, hypertension or CVD, smoking, physical activities, diet, lipid profile, fasting blood glucose (FGlu), Hemoglobin A1c (HbA1C), blood pressure (BP) and body mass index (BMI). Patients are recommended to be assessed with increased frequency if they are being treated with antipsychotic medication [23, 24]. Although most of the psychiatrists and medical professionals within mental health hospitals are aware of the high risk of comorbid metabolic syndromes for those patients with SMI, evidence exists about the suboptimal management of comorbid metabolic syndromes among patients with SMI due to various reasons such as the focused management of psychiatric symptoms and adherence to psychiatric treatment [25, 26]. In some countries including Singapore, patients with SMI in Singapore were referred to family physicians or other general hospitals for further treatment once they have been diagnosed with comorbid metabolic syndromes [27–29].

Substantial evidence has demonstrated that patients with mental illnesses are at a higher risk for hospital readmission due to poor physical health conditions compared to those without mental illness [30–32]. Large body of studies also supported the hypothesis that patients with mental illness are at increased risk of psychiatric readmission if they had co-occurring medical condition [33, 34]. However, it remains unclear whether in-hospital measurement of cardiovascular risk factors such as blood test of metabolic risk factors and blood pressure during the period of psychiatric admission have essential influence on the subsequent psychiatric readmission. To address this gap, we proposed a retrospective study using electronic health records (EHRs) of in-hospital patients with diagnosis of SMI and with comorbid DM, hyperlipidemia or hypertension who requires more intensive attention of CVD risk factor management than those patients without comorbid DM, hyperlipidemia or hypertension. We hypothesize that poorer control of the cardiovascular metabolic risk factors for in-hospital patients with SMI are associated with higher risks of psychiatric readmission.

## Methods

### Design, settings and study sample

This is a retrospective cohort study. We collected the data from EHRs of patients in the Institute of Mental Health (IMH) from 01 January 2014 to 31 January 2019. The inclusion criteria were 1) Patients with at least one primary diagnosis of schizophrenia, major depression disorder or bipolar disorder. The details of the diagnosing code and subtypes of diagnosis are listed in Supplementary table 1. 2) Patients with at least one psychiatric admission during the study period. 3) Patients with history of DM, hyperlipidemia or hypertension before the first admission. The inclusion criteria were only applied for patients with acute admissions and all forensic cases were excluded. All methods in this study were carried out in accordance with guidelines and regulations stated in the local ethnic committee-Domain Specific Review Board (DSRB) Investigator’s manual with patient’s informed consent being waived.

### Data collection

We extracted relevant patient information from electronic medical records using a data collection form. This included socio-demographic information such as age, gender and ethnicity; clinical information such as medical history, diagnosis, hospitalization admission date, admission type, admission diagnosis, discharge date, discharge diagnosis, medicine prescription including antipsychotics, antidepressants, mood stabilizers, drugs that control blood pressure, blood glucose and lipids, date of psychotherapies and electroconvulsive therapies (ECT), blood tests including high density lipoprotein (HDL), low density lipoprotein (LDL), fasting glucose and HbA1C, blood pressure and BMI. For patients with multiple records of blood tests and parameters, we chose to present the result closest to the first discharge date.

### Definitions

We obtained the blood tests’ results from the laboratory information systems and the records of BP and BMI from the patients’ case notes. According to the current practicing guidelines in National Healthcare Group in Singapore [35], among patients with a diagnosis of DM, HDL ≥ 1.0 mmol/l, LDL < 2.6 mmol/L, FGlu < 7.0 mg/L, HbA1C < 7.0%, SBP < 140 mmHg, DBP < 80 mmHg, and BMI < 23 kg/m^2^ were considered as normal testing of their CVD risk factors. Among patients without a comorbid diagnosis of DM (i. e. hyperlipidemia or hypertension), HDL ≥ 1.0 mmol/l, LDL < 4.1 mmol/L, FGlu < 6.0 mg/L, HbA1C < 6.0%, SBP < 140 mmHg, DBP < 90 mmHg and BMI < 23 kg/m^2^ were considered as normal testing of their cardiovascular metabolic risk factors. Otherwise, the risk factors were considered as “abnormal test”. Suboptimal monitoring of CVD risk factors was defined as without performing the above tests. Optimal monitoring of CVD risk factors was defined as with performing the above tests regardless of the testing outcome.

Considering some patients were lacking records of blood test or screening of BP and BMI after admission, we also utilized the 2011 European guideline of metabolic risk factor monitoring by De Hert, M. et al. [24] to pick up patients’ records during a window period of CVD risk factor screening before the index hospitalization, i.e. within one year for lipid profile, FGlu, HbA1C and within three months for BP and BMI.

### Statistical analysis

We used descriptive statistics for sociodemographic and clinical characteristics. Numerical variables were presented as mean ± standard deviation (SD) and categorical variables were presented as count and percentage (%). Cox regression model (proportional hazard model) was utilized to calculate the association between risk predictors and clinical outcomes. The major risk predictors (variables of interest) were blood tests, BP and BMI which were dichotomized into “normal tests vs abnormal test” or “optimal monitoring vs suboptimal monitoring” as defined previously. For risk prediction only patients’ information before and during first admission were included as predictor variables. The outcome was time to the first readmission. Calculated hazard risks (HRs) were adjusted for patients’ socio-demographics and clinical characteristics including primary diagnosis, admission type, medicine prescription, behavior therapy and ECT therapy during first hospitalization period. Statistical significance was accepted at *p* < 0.05 level for all tests. Data were analyzed by SAS 6.0 and STATA (*version 15.0*).

## Results

A total of 5,256 patients were included in this study and their socio-demographics are presented in Table 1. The mean age was 56 ± 14 (mean ± SD) years and 47.3% were female. 73.6% of recruited patients were Chinese, 11.4% were Malay and 10.9% were Indian. 64.2% of the patients were diagnosed with schizophrenia, 25.7% were diagnosed with major depression disorder and 10.2% were diagnosed with bipolar disorder. There were 2539 (48.3%) patients with comorbid diagnosis of hypertension, 3142 (59.8%) with comorbid DM and 4221 (80.3%) with comorbid hyperlipidemia.

**Table 1:** Patients’ socio-demographics and clinical characteristics (*n* = 5256)

Patient characteristics		Count	%
Age (mean ± SD)		56 ± 14	
Gender	Female	2485	47.3
	Male	2771	52.7
Ethnicities	Chinese	3870	73.6
	Malay	600	11.4
	Indian	575	10.9
	Caucasian	21	0.4
	Others	190	3.6
Religion	Buddhism	366	7
	Christine	221	4.2
	Hinduism	99	1.9
	Islam	243	4.6
	Taoism	30	0.6
	Free thinker	160	3
	No mapping	4137	78.7
Marital Status	Married	1515	28.8
	Divorced	210	4
	Single	1702	32.4
	Widowed	52	1
	No mapping	1777	33.8
Primary diagnosis	Schizophrenia	3373	64.2
	Depression	1349	25.7
	Bipolar disorder	534	10.2
	Hypertension	2539	48.3%
	Diabetes	3142	59.8%
Cardiovascular diagnosis	Hyperlipidemia	4221	80.3%
	Any 2 combined	1560	29.7%
	Triple high	1543	29.4%
Medicine prescription	Antipsychotics	3729	70.9
	Antidepressant	2516	47.9
	Mood stabilizer	3021	57.5
	CVD protection	203	3.9
	Lowering blood glucose	1013	19.3
	Lowering blood lipid	2116	40.3
	Lowering blood pressure	1367	26
	Others	3921	74.6
Behavior therapies	Psychotherapy	187	3.6
	Structured educational therapy	1515	28.8
	Structured group therapy	712	13.5
	Structured recreational therapy	1828	34.8
Other treatment	Electroconvulsive therapy	110	2.1

*SD* standard error, *CVD* cardiovascular disease

**Fig. 1:**
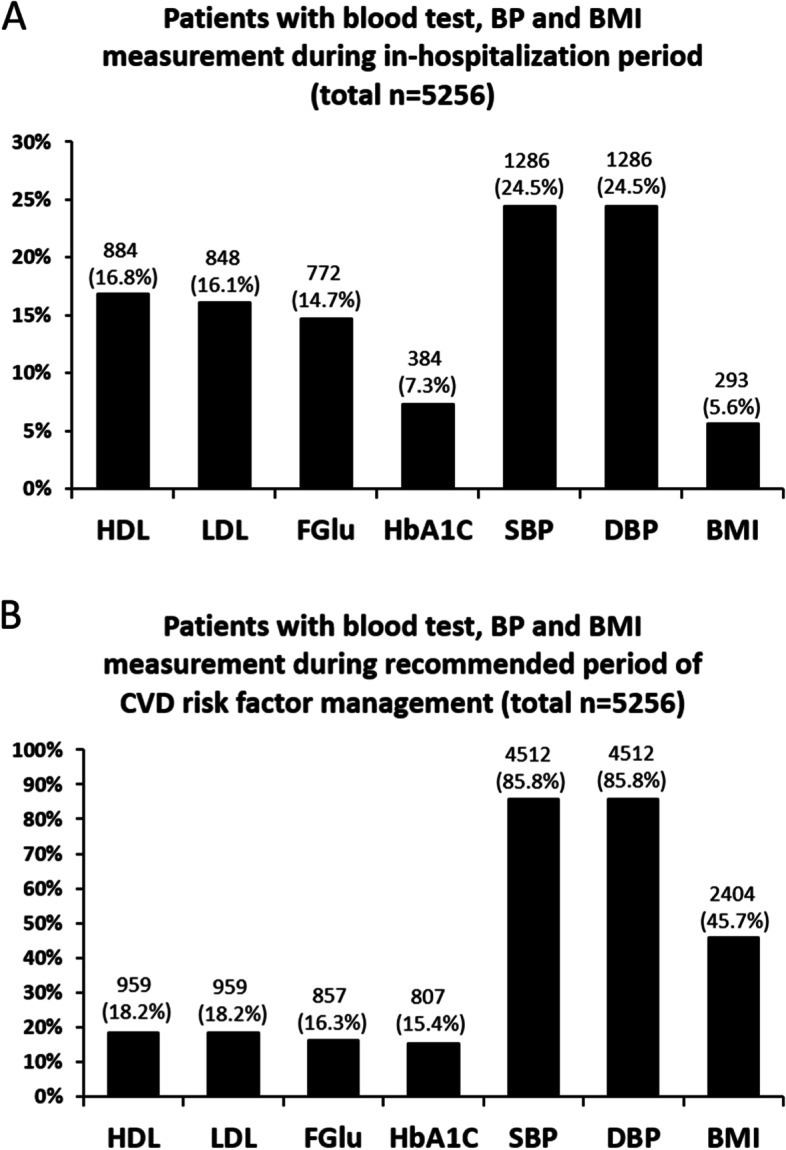
Percentage of patients with blood test, BP measurement and BMI measurement during hospitalization period or recommended period of CVD risk factor management

For blood tests during admission, HDL and LDL were performed for 16.8% and 16.1% of the total patients, FGlu for 14.7% of the total patients and HbA1C for 7.3% of the total patients. For parameters, BP was recorded for 24.5% and BMI for 5.6% of the total patients (Fig. 1A). Within the recommended window period of CVD risk factor screening, 18.2% of total patients had blood test for HDL/LDL, 16.3% for FGlu, 15.4% for HbA1C, 85.8% for measurement of BP and 45.7% with measurement for BMI. (Fig. 1B).

During the in-hospitalization period, compared to patients with normal tests, patients with abnormal tests of HDL and DBP were associated with higher hazard risks of subsequent psychiatric readmission (adjusted HR, 1.37 [95% CI, 1.03–1.83] for HDL and adjusted HR, 1.32 [95% CI, 1.04–1.67] for DBP, Table 2). Compared to patients with optimal CVD risk factor monitoring during the hospitalization period, patients with suboptimal monitoring of HDL, LDL, FGlu, SBP, DBP and BMI were associated with higher hazard risks of subsequent psychiatric readmission (adjusted HR, 1.67 [95% CI, 1.47–1.89] for HDL; adjusted HR, 1.68 [95% CI, 1.48–1.91] for LDL; adjusted HR, 1.69 [95% CI, 1.48–1.93] for FGlu; adjusted HR, 1.66 [95% CI, 1.49–1.85] for SBP; adjusted HR, 1.66 [95% CI, 1.49–1.85] for DBP and adjusted HR, 1.37 [95% CI, 1.12–1.67] for BMI).

**Table 2:** Association of in-hospital blood tests/parameters with time to 1^st^ psychiatric readmission

CVD risk factors	Optimal		Suboptimal	Readmission event				Abnormal vs Normal			Suboptimal vs Optimal		
	Normal test [n (%)]	Abnormal test [n (%)]	No screening [n (%)]	Normal test [n (%)]	Abnormal test [n (%)]	No screening [n (%)]		HR	95% CI	P value	HR	95% CI	P value
HDL	706 (13.4%)	178 (3.4%)	4372 (83.2%)	215 (30.5%)	72 (40.4%)	2078 (47.5%)	Crude	1.45	1.11 – 1.90	0.006^b^	1.61	1.42–1.82	< 0.001^c^
							Adjusted^a^	1.37	1.03 – 1.83	0.030^b^	1.67	1.47–1.89	< 0.001^c^
LDL	553 (10.5%)	295 (5.6%)	4408 (83.9%)	201 (32.4%)	48 (31.8%)	2116 (47.2%)	Crude	0.9	0.70 – 1.15	0.400	1.61	1.42–1.82	< 0.001^c^
							Adjusted^a^	0.93	0.72—1.20	0.572	1.68	1.48–1.91	< 0.001^c^
FGlu	621 (11.8%)	151 (2.9%)	4484 (85.3%)	246 (28.5%)	167 (39.6%)	1952 (49.2%)	Crude	0.97	0.71—1.34	0.874	1.61	1.41–1.84	< 0.001^c^
							Adjusted^a^	1.08	0.78—1.50	0.636	1.69	1.48–1.93	< 0.001^c^
HbA1C	281 (5.3%)	103 (2.0%)	4872 (92.7%)	32 (29.6%)	69 (37.3%)	2264 (45.6%)	Crude	0.92	0.66 – 1.29	0.639	1.01	0.87–1.18	0.867
							Adjusted^a^	0.84	0.59—1.19	0.325	1.07	0.91–1.25	0.428
SBP	864 (16.4%)	422 (8.0%)	3970 (75.5%)	185 (33.5%)	90 (30.5%)	2090 (47.4%)	Crude	1.46	1.20 – 1.78	< 0.001^c^	1.76	1.59–1.96	< 0.001^c^
							Adjusted^a^	1.19	0.95—1.50	0.129	1.66	1.49–1.85	< 0.001^c^
DBP	942 (17.9%)	344 (6.5%)	3970 (75.5%)	128 (45.6%)	45 (43.7%)	2192 (45.0%)	Crude	1.57	1.28 – 1.93	< 0.001^c^	1.76	1.59–1.96	< 0.001^c^
							Adjusted^a^	1.32	1.04 – 1.67	0.022^b^	1.66	1.49–1.85	< 0.001^c^
BMI	108 (2.1%)	185 (3.5%)	4963 (94.4%)	269 (28.6%)	144 (41.9%)	1952 (49.2%)	Crude	1.35	0.89 – 2.05	0.162	1.43	1.17–1.74	< 0.001^c^
							Adjusted^a^	1.37	0.90 – 2.08	0.148	1.37	1.12–1.67	0.002^b^

*CVD* cardiovascular disease, *HDL* high-density dyslipidemia, *LDL* low-density dyslipidemia, *FGlu* fasting glucose, *HbA1C* hemoglobin A1C, *SBP* systolic blood pressure, *DBP* diastolic blood pressure, *HR* hazard ratio, *CI* confidence interval, *ECT* electroconvulsive therapy

^a^Adjusted for age, gender, ethnicities, religion, marital status, primary diagnosis, admission type, medicine prescription, behavior therapy and ECT treatment

^b^*P* < 0.05

^c^*P* < 0.01

During the recommended period of CVD risk factor management, compared to patients with normal tests, patients with abnormal tests of HbA1C were associated with higher hazard risks of subsequent psychiatric readmission (adjusted HR, 12.2 [95% CI, 2.55–58.38], Table 3). Compared to patients with optimal monitoring, patients with suboptimal monitoring of HDL, LDL, FGlu, SBP and DBP were associated with higher hazard risks of psychiatric readmission (adjusted HR, 1.74 [95% CI, 1.54–1.97] for HDL; adjusted HR, 1.74 [95% CI, 1.54–1.97] for LDL; adjusted HR, 1.75 [95% CI, 1.54–1.99] for FGlu; adjusted HR, 2.07 [95% CI, 1.87–2.30] for HDL and adjusted HR, 2.07 [95% CI, 1.87–2.30] for DBP, Table 3).

**Table 3:** Association of blood tests/parameters during recommended period of CVD risk factor management with time to 1^st^ psychiatric readmission

**CVD risk factors**	**Optimal**		**Suboptimal**	**Readmission event rate**				**Abnormal vs Normal**			**Suboptimal vs Optimal**		
	Normal test [n (%)]	Abnormal test [n (%)]	No screening [n (%)]	Normal test [n (%)]	Abnormal test [n (%)]	No screening [n (%)]		HR	95% CI	P value	HR	95% CI	P value
HDL	816 (15.5%)	143 (2.7%)	4297 (81.8%)	252 (30.9%	51 (35.7%)	2023 (47.1%)	Crude	1.21	0.90–1.64	0.209	1.63	1.44–1.84	< 0.001^c^
							Adjusted^a^	0.51	0.12–2.24	0.371	1.74	1.54–1.97	< 0.001^c^
LDL	624 (11.9%)	335 (6.4%)	4297 (81.8%)	217 (31.4%)	53 (31.7%)	2056 (46.7%)	Crude	0.93	0.73–1.18	0.530	1.63	1.44–1.84	< 0.001^c^
							Adjusted^a^	0.66	0.23–1.89	0.439	1.74	1.54–1.97	< 0.001^c^
FGlu	690 (13.1%)	167 (3.2%)	4399 (83.7%)	1086 (38.7%)	723 (42.4%)	517 (69.5%)	Crude	1.01	0.75–1.37	0.942	1.63	1.43–1.85	< 0.001^c^
							Adjusted^a^	1.38	0.39–4.83	0.618	1.75	1.54–1.99	< 0.001^c^
HbA1C	619 (11.8%)	188 (3.6%)	4449 (84.6%)	320 (47.7%)	869 (50.1%)	1137 (39.9%)	Crude	1.26	0.99–1.62	0.063	1.13	1.01–1.27	0.036^b^
							Adjusted^a^	12.2	2.55–58.38	0.002^c^	1.10	0.97–1.25	0.122
SBP	2808 (53.4%)	1704 (32.4%)	744 (14.2%)	202 (32.4%)	101 (30.1%)	2023 (47.1%)	Crude	1.11	1.01–1.22	0.024^b^	2.37	2.15–2.61	< 0.001^c^
							Adjusted^a^	1.77	0.61–5.15	0.295	2.07	1.87–2.30	< 0.001^c^
DBP	3110 (59.2%)	1402 (26.7%)	744 (14.2%)	239 (38.6%)	86 (45.7%)	2001 (45.0%)	Crude	1.28	1.16–1.41	< 0.001^c^	2.37	2.15–2.61	< 0.001^c^
							Adjusted^a^	1.07	0.36–3.17	0.903	2.07	1.87–2.30	< 0.001^c^
BMI	671 (12.8%)	1733 (33.0%)	2852 (54.3%)	1168 (37.6%)	641 (45.7%)	517 (69.5%)	Crude	1.06	0.93–1.21	0.363	0.75	0.69–0.82	< 0.001^c^
							Adjusted^a^	0.56	0.17–1.87	0.347	0.92	0.85–1.00	0.055

*CVD* cardiovascular disease, *HDL* high-density dyslipidemia, *LDL* low-density dyslipidemia, *FGlu* fasting glucose, *SBP* systolic blood pressure, *DBP* diastolic blood pressure, *HR* hazard ratio, *CI* confidence interval, *ECT*, electroconvulsive therapy

^a^Adjusted for age, gender, ethnicities, religion, marital status, primary diagnosis, admission type, medicine prescription, behavior therapy and ECT treatment

^b^*P* < 0.05

^c^*P* < 0.001

## Discussion

The key finding of this study is that among in-hospital patients with SMI, poor monitoring (missing screening) of metabolic risk factors was associated with higher risks of psychiatric readmission. i. e. a shorter time to psychiatric readmission. To our knowledge so far, this is the first study reporting the association of psychiatric in-hospital measurement of cardiovascular metabolic risk factors with psychiatric readmission.

The regular screening and management of metabolic risk factors is essential to decrease the incidence of adverse cardiovascular events and mortality among patients with SMI. In our study, large proportion of patients with SMI and with comorbid cardiovascular diseases did not receive screening of cholesterol, glucose, BP or BMI during in-hospitalization period or a relative longer recommended window period of screening. One possible reason is due to missing data entry. However, missing data is unlikely to completely account for the relatively large proportion of patients lacking CVDs risk factor measurement during in-hospital treatment period. Another possibility is that the treating psychiatrist in our hospital did not routinely screen the patients for comorbid metabolic biomarkers and this is not unusual. Previous studies reported that about a high percentage till 70% of patients taking secondary generation antipsychotics remained unscreened for risks factors of diabetes or dyslipidemia [36–38]. The barriers of screening were most likely due to various logistic reasons including the heavy burden of psychiatric symptoms, insufficient staff, wait times for medical follow-up and difficulties coordinating with off-campus physicians etc. [25, 26]. However, implementation of a complete set of metabolic risk factor screening for those patients with SMI and high risk of comorbid CVDs is feasible [39]. One effective tool for improving rates of screening is the adoption of an in-house medical service from family physicians.

The adoption of in-house medical services was supported by several randomized clinical trials to screen and treat comorbid physical illness among patients with SMI, which proved to be effective in terms of the improvement of patient quality of life and a reduction of cardiovascular risks [40, 41]. However, we have known that large quantities of patients were absent from the follow-up appointment with on-site primary care physicians after being discharged from the mental hospital [29]. Further implementation measurements, therefore, are needed to reinforce the screening and subsequent treatment of cardiovascular metabolic risk factors by on-site family physicians within mental hospital.

Comorbid physical illness is one emerging risk factor for psychiatric readmission. Large number of studies has proven the high incidence of comorbid physical illness among patients with SMI. The association of physical conditions with psychiatric readmission can vary according to the nature of mental disorders, characteristics of study population, applied concept of comorbidity, and study protocol [34]. Reported from a study among patients with mental and/or substance use disorder (SUD), comorbid diagnosis of DM or hypertension was associated with increased risk of psychiatric readmission within 30 days after discharge [42]. Patients with SMI were also reported to have an increased risk of psychiatric readmission within seven years after discharge if they had higher Charlson comorbidity index scores [43]. In our study, instead of comparing the patients with and without history of comorbid physical illness, we examined the impact of in-hospital blood tests of CVD risk factors or BP on the risks of psychiatric readmission among patients with known history of CVDs.

We found that abnormal testing and the poor monitoring of CVD blood risk factors and BP were associated with higher risks of psychiatric readmission after adjustment for socio-demographics, drugs dispensed and other psychiatric treatment. Among the targeted CVD risk factors, blood lipids and blood pressure were significant contributors of these association as abnormal tests/suboptimal monitoring of HDL and DBP were consistently associated with increased risks of psychiatric readmission after adjustment for socio-demographics and other clinical characteristics. The central nervous system mechanisms of the action of these blood parameters or BP may account for the observed association between blood lipids or blood pressures and psychiatric readmission. Human studies have yielded promising results about the role of cholesterol or cholesterol-containing molecules in the prognosis of SMI. Although LDL is one of the major blood lipids screened by physicians to monitor the metabolic conditions, a considerable body of research has demonstrated that compared with healthy people, MDD patients including those protracted cases who lack remission often exhibit a decrease in HDL [44, 45]. Dyslipidemia and lower level of plasma HDL were also reported to be associated with acute-phase schizophrenia [46]. Another index of metabolic syndromes, BP, was proposed to explain the associations between psychopathology and CVDs among patients with depressive symptoms while the direction of those associations was contradictory [47–50] and may be confounded by the use of antidepressants [51]. Pulse pressure was reported to be significantly correlated with cognitive impairment, which was a core feature of schizophrenia [52]. Therefore, our examinations of the relationship between blood lipids/blood pressure and psychiatric readmission may provide insight into understanding the common biological mechanisms underpinning CVDs, and later psychiatric relapse.

Our study provided the evidence of cardiovascular metabolic risk factors measurement, particularly HDL or BP, as the risk predictors of psychiatric readmission among patients with SMI. Therefore, any treatment to monitor the serum lipid and blood pressure would be beneficial to minimize the psychiatric illness burden such as reducing the risks of psychiatric admission. Indeed, it was recently reported that drugs in common use for metabolic health problems such as statins, calcium channel antagonists and metformin were associated with reduced rates of psychiatric admission and self-harm in individuals with SMI [53]. Given the massive illness burden of SMI, future randomized controlled studies are needed to validate the impact of the CVD risk factors screening and treatment during in-hospitalization period on subsequent psychiatric readmissions.

Our study is limited by the retrospective study design. Psychiatric hospitalization is an important means of stabilizing deteriorating psychiatric conditions, re-establishing discontinued regimen of prescribed medication and offering help to transit individuals to outpatient and community-based mental health services. Many individuals with SMI experience multiple psychiatric readmissions [5]. A number of factors have been proposed to be associated with psychiatric readmission among patients with SMI including the patients’ previous number of hospitalization, which had been consistently reported to be the highest risk factor for readmission [54, 55] and the post-discharge factors, such as poor social support [56]. Therefore, the screening of cardiovascular metabolic risk factors at or before admission and after patients had been discharged from hospital might overstate the importance of monitoring the cardiovascular metabolic risk factors while the patient was in hospital, although those unmeasured confounders beyond the in-hospitalization period would be unlikely to fully explain our findings. In addition, we didn’t collect other CVD related risk factors during hospitalization period such as smoking, drinking status and the severity of psychiatric symptoms. The latter was highly associated with the risks of psychiatric readmission which could be attributed to symptoms related drug prescription such as clozapine.

In summary, in-hospital poor measurement of cardiovascular metabolic risk factors was found to be associated with increased risks of psychiatric readmission among patients with SMI. Our finding implies that the medical professionals including both psychiatrists and family physicians in mental hospital may need to be more active in the screening and monitoring of CVD risk factors for in-hospital patients with SMI. Moreover, the observed association between components of cardiovascular metabolic risk factors with psychiatric readmission indicated that those CVD risk factors, such as blood lipid and blood pressure, could be new targets of therapeutic intervention to improve psychiatric care.

## Supplementary Information

**Additional file 1:** **Supplementary Table 1**. Group of diagnosis.

## Data Availability

The data that support the findings of this study is not publicly available and only accessible from the corresponding author (Dr Xiao Wei Tan xiaowei_tan@imh.com.sg) on reasonable request via approval by the Institutional Research Review Committee and the National Healthcare Group Domain Specific Review Board.

## Abbreviations

SMI: Severe mental illness
MDD: Major depression disorder
CVD: Cardiovascular disease
DM: Diabetes mellitus
FGlu: Fasting blood glucose
HbA1C: Hemoglobin A1c
SBP: Systolic blood pressure
DBP: Diastolic blood pressure
BMI: Body mass index
EHR: Electronic health record
ECT: Electroconvulsive therapy
HDL: High density lipoprotein
LDL: Low density lipoprotein
SD: Standard deviation
HR: Hazard risk
CI: Confidence interval
