# Supplementary material for: Association of cardiovascular metabolic risk factor measurements with psychiatric readmission among in-hospital patients with severe mental illness: a retrospective study
Source: BMC Psychiatry. 2022 Jan 18;22:43. doi: 10.1186/s12888-022-03704-w (PMC8767705; doi:10.1186/s12888-022-03704-w)
Supplement: Supplementary file 1 — Additional file 1: Supplementary Table 1. Group of diagnosis. [file 12888_2022_3704_MOESM1_ESM.docx]

Supplementary Table 1: Group of diagnosis

| **No.** | **Diagnosis Description** | **Grouped diagnosis** |
| --- | --- | --- |
| 1 | Other schizoaffective disorders | Schizophrenia |
| 2 | Other schizophrenia |  |
| 3 | Paranoid schizophrenia |  |
| 4 | Residual schizophrenia |  |
| 5 | Schizoaffective disorder, depressive type |  |
| 6 | Schizoaffective disorder, manic type |  |
| 7 | Schizoaffective disorder, unspecified |  |
| 8 | Schizoaffective disorders |  |
| 9 | Schizophrenia |  |
| 10 | Schizophrenia, unspecified |  |
| 11 | Schizotypal disorder |  |
| 12 | Simple schizophrenia |  |
| 13 | Hebephrenic schizophrenia |  |
| 1 | Depressive episode | Depression |
| 2 | Depressive episode, unspecified |  |
| 3 | Depressive episode, unspecified, not specified as arising in the postnatal period |  |
| 4 | Recurrent depressive disorder |  |
| 5 | Recurrent depressive disorder, current episode moderate |  |
| 6 | Recurrent depressive disorder, current episode severe with psychotic symptoms |  |
| 7 | Recurrent depressive disorder, current episode severe without psychotic symptoms |  |
| 8 | Recurrent depressive disorder, unspecified |  |
| 9 | Mild depressive episode |  |
| 10 | Mild depressive episode, not specified as arising in the postnatal period |  |
| 11 | Mixed anxiety and depressive disorder |  |
| 12 | Severe depressive episode with psychotic symptoms |  |
| 13 | Severe depressive episode with psychotic symptoms, not specified as arising in the postnatal period |  |
| 14 | Severe depressive episode without psychotic symptoms |  |
| 15 | Severe depressive episode without psychotic symptoms, not specified as arising in the postnatal period |  |
| 1 | Bipolar affective disorder | Bipolar disorder |
| 2 | Bipolar affective disorder, current episode manic with psychotic symptoms |  |
| 3 | Bipolar affective disorder, current episode manic without psychotic symptoms |  |
| 4 | Bipolar affective disorder, current episode mild or moderate depression |  |
| 5 | Bipolar affective disorder, current episode mixed |  |
| 6 | Bipolar affective disorder, unspecified |  |
